# Willingness to pay for family education and counselling services provided by critical care advanced practice nurses

**DOI:** 10.1111/ijn.12782

**Published:** 2019-09-12

**Authors:** Chung Mee Ko, Chin Kang Koh, Sangho Kwon

**Affiliations:** ^1^ College of Nursing Sungshin Women's University Seoul South Korea; ^2^ College of Nursing Seoul National University Seoul South Korea; ^3^ Department of Tax and Accounting Shingu College Seongnam South Korea

**Keywords:** advanced practice nursing, contingent valuation method, counselling, critical care, education, willingness to pay

## Abstract

**Aim:**

The aim of this study was to estimate the economic value of a family education and counselling service provided by critical care advanced practice nurses in South Korea utilizing a contingent valuation approach.

**Methods:**

A double‐bounded dichotomous choice contingent valuation method was utilized to estimate the public's willingness to pay value for an education and counselling service provided by critical care advanced practice nurses. A web‐based self‐administered survey was conducted.

**Results:**

Median willingness to pay was 43 112 Korean won (35 US dollars). Higher income and younger age were associated with higher willingness to pay.

**Conclusion:**

This study captured the economic value of an education and counselling service provided by critical care advanced practice nurses that is not on the benefit list under the fee‐for‐service system of the Korean National Health System. Policy makers should consider including such services in the health care system.

## SUMMARY STATEMENT

What is already known about this topic?

- Patients in intensive care units and their family members need education and counselling.
- Nurses and physicians recognize the importance of the education and counselling services provided by critical care advanced practice nurses.
- No studies have evaluated the economic value of the family education and counselling services provided by critical care advanced practice nurses.

What this paper adds?

- The Korean people in this study recognized the economic value of a service provided by critical care advanced practice nurses.
- Income and age were found to be factors related to the economic value of the education service provided by critical care advanced practice nurses.

The implications of this paper:

- This study captured the economic value of an education and counselling service provided by critical care advanced practice nurses, the fee for which is not reimbursed by the current Korean health care fee‐for‐service system.
- Policy makers should consider the public perception of the economic value of the education and counselling services provided by critical care advanced practice nurses when determining the benefits and the prices of services included in the Korean National Health Insurance System.

## 1 INTRODUCTION

Advanced practice nursing education at the master's level was introduced in South Korea in 2003. According to the Korean Nurses Association, “Advanced practice nurses serve patients with superior nursing services based on extensive knowledge along with professional nursing techniques and nursing related studies in his or her own specialized field” (Korean Nurses Association, 2013). Professional clinical nursing practice (direct care), education and counselling, research, leadership, consultation, and collaboration are core advanced practice nurse (APN) competencies endorsed by the Korean Accreditation Board of Nursing Education (Korean Accreditation Board of Nursing Education, 2004).

Thirteen APN specialty areas are authorized by the Medical Service Act in South Korea (Korean Nurses Association, 2013), and critical care advanced practice nursing is one of these. In South Korea, there is a shortage of board‐certified full‐time intensivists in intensive care units (ICUs) (Lee et al., 2016), and only 30% of ICUs have at least one full‐time intensivist (Lim, Kwak, Suh, & Koh, 2015. Thus, critical care APNs are expected to play an active role in ICUs (Kim et al., 2009).

Family members of patients in ICUs have reported their need for education and counselling. In a review of 30 articles, Al‐Mutair, Plummer, O'Brien, and Clerehan (2013) found that family members of patients in ICUs gave high priority to the needs for assurance and information. The need for assurance is the family's need to be reassured about the health status of the patient and the patient's outcome, while the need for information relates to family members seeking knowledge about various issues associated with the patient's condition (Al‐Mutair, Plummer, O'Brien, & Clerehan, 2013). However, these needs for assurance and information are inconsistently met (Omari, 2009). Moreover, it has been reported that family members have complaints about the lack of communication with physicians and nurses in ICUs, which causes family members to feel helpless (Lee & Yi, 2017).

Structured education programmes aid in meeting the needs for assurance and information (Kynoch, Chang, Coyer, & McArdle, 2016. Kynoch et al. (2016) also reported that educational interventions might reduce family members' anxiety and distress and decrease the number of phone calls from family members to ICUs. In addition, family meetings to discuss the patient's condition, care goals, and preferences improve family members' satisfaction with the ICU stay and increase their feeling that they are in control (Wu, Ren, Zinsmeister, Zewe, & Tuite, 2016).

In ICUs, it is recommended that health professionals provide family members with regular updates and support to reduce their stress and anxiety (Davidson et al., 2007). However, physicians' availability is limited, and some nurses have difficulty maintaining consistent communication with the family about decision making (Kalocsai et al., 2018). Although there is very limited evidence regarding the education and counselling roles of APNs in ICUs, APNs in acute care settings help to improve family satisfaction by being available to deliver updates on the patient's status and teach and provide education, and they provide more face‐to‐face care than physicians (Crowe, 2014). A qualitative study found that parents of children in a neonatal ICU perceived that APNs helped them feel reassured and assisted them by translating information (Beal & Quinn, 2002).

In a study in South Korea, physicians, nurses, and APNs ranked the education and counselling role as the most important role of APNs (Cho, Cho, Kwon, Seo, & Baek, 2011). Nurses in ICUs also recognize that providing education and counselling for patients and their family members is an essential role of critical care APNs (Lee et al., 2007). However, APNs in ICUs mainly spend their time providing direct clinical care, such as tracheostomy management, artificial airway management, BiPAP application, ventilator and weaning management, central line insertion, nasogastric tube insertion, wound dressing, and continuous renal replacement equipment management (Kim, Kim, Yi, & Kang, 2011; Kim et al., 2009). Even when an education programme is provided to family members, the APN's contribution is not visibly recognized by the health care institution (Kim, Kim, Yi, & Park, 2011).

Although ICU patients and their family members have a need for education and counselling and even though physicians and nurses recognize the importance of education and counselling provided by APNs, health care institutions do not particularly value this role of critical care APNs (Kim et al., 2011). One main reason for this is that most APN services are not directly reimbursed by the Korean National Health Insurance System (Kim et al., 2009).

The payment system of the Korean National Health Insurance Service is mainly based on a fee‐for‐service model, in which a fee is charged for each treatment activity that is performed (National Health Insurance Service, 2015). In the ICUs, the majority of activities that critical care APNs perform involving treatment, such as intubation, suctioning, BiPAP application, continuous renal replacement equipment management, nasogastric tube insertion, and wound dressing, are reimbursed under the fee‐for‐service model of the National Health Insurance System (Kim et al., 2009). However, the costs of the education and counselling services provided by critical care APNs in ICUs are not reimbursed. Anecdotally, a critical care APN in a neonatal ICU reported that she provided an educational programme for parents to relieve their anxiety, but the hospital was not reimbursed for any of the costs of the programme (Kim et al., 2011). Thus, the lack of recognition by the nationally financed insurance programme regarding the economic value of the education and counselling services that critical care APNs provide to family members is a major barrier to the provision of such services. Nevertheless, no studies have evaluated the economic value of these APN services.

The contingent valuation method (CVM) is a nonmarket valuation method that can be used to estimate economic values. Contingent valuation is “reflected in expressions of the willingness to pay (WTP) for potential benefits or for the avoidance of their loss” (OECD, 2008, p. 102). Initially, this method was mainly utilized to estimate ecological environmental benefits. However, it is currently widely used for all kinds of nonmarket resources, such as cultural resources, safety and health resources, and public information (Kwon & Ryu, 2013).

Many studies have estimated the economic value of health care resources using CVM. Respondents are asked “to think about the contingency of an actual market existing for a programme or health benefit and the maximum they would be willing to pay” (Drummond, Sculpher, Caxton, Stoddart, & Torrance, 2015, p. 184). CVM has also been utilized to evaluate diagnostic laboratory tests in several studies (Lin, Cangelosi, Lee, & Neumann, 2013). Van den Berg, Brouwer, van Exel, and Koopmanschap (2005) used CVM to determine the monetary value of a hypothetical additional hour of informal care for persons with rheumatoid arthritis, and Martín‐Fernández et al. (2013) utilized CVM to evaluate the monetary value of nursing consultations at primary care centres.

With regard to the survey method, this study used an online survey panel. The U.S. National Oceanic and Atmospheric Administration's (NOAA's) guidelines for CVM survey designs recommend face‐to‐face interview surveys. However, the guidelines were published in 1993 when the Internet infrastructure and survey software were not yet established. Thus, online surveys were not assessed as an option at that time. Recently, online survey panels have been utilized in several CVM studies (Sueki, 2013; Sueki, 2016; Yasunaga, Ide, Imamura, & Ohe, 2006. Moreover, a study using CVM compared the web‐based survey mode and the face‐to‐face interview survey mode and found that WTP values were statistically not different between two samples using the two different survey modes (Nielsen, 2011). This result suggests that an online survey is suitable for a CVM study. Moreover, this survey mode offers the advantage of saving researchers' time, thus reducing cost (Wright, 2005).

## 2 METHODS

### 2.1 Aim

The aim of this study was to estimate the economic value of the family education and counselling services provided by critical care APNs in South Korea utilizing a contingent valuation approach. In order to achieve this aim, this study attempted to establish a median WTP value for this type of APN service and examine factors associated with the public's WTP value.

### 2.2 Study design

A double‐bounded dichotomous‐choice CVM was utilized to estimate the public's WTP value for an education service provided by critical care APNs. A web‐based self‐administered survey was administered.

### 2.3 Participants

A total of 615 participants aged 19 to 49 years participated in this study. As a web‐based survey was used in this study, young and middle‐aged adults less than 50 years old who had more access to the Internet and were more familiar with web‐based surveys were included in this study. The online survey was administered in November and December of 2016 by a major Internet survey company. This research survey company has the largest panel (more than 1.2 million panellists) in South Korea and has worked with several governmental and nongovernmental research projects. The survey company selected 615 samples randomly from the registered panel members on the basis of age, gender, and region. There were four types of survey questions based on the first bid price. One of the four types was randomly sent to each participant.

A sample size of at least 500 is recommended to estimate median WTP (Hanemann & Kanninen, 1999). However, this number is a recommendation, and in a review of WTP studies, 18 of 38 studies had a sample size less than 500 (Meyerhoff, Mørkbak, & Olsen, 2014).

### 2.4 Questionnaire

The questionnaire was developed by the researchers of this study. This CVM study questionnaire included the following sections: (a) the hypothetical scenario, (b) the elicitation method (a double‐bounded dichotomous choice) to ask about respondents' WTP, and (c) socio‐economic information (Klose, 1999; Sueki, 2013; Yasunaga et al., 2006).

First, a hypothetical scenario describing an education and counselling service to be offered was developed by the researchers of this study on the basis of the literature. Then, this hypothetical scenario was reviewed by three critical care APNs. The education and counselling service in the hypothetical scenario consisted of information about the patient's health, the ICU environment, and advanced care planning. This service took 30 minutes each time it was offered. Because this study aimed to determine the general public's perception, the majority of the sample had no experience with ICU admissions. Therefore, the scenario described the ICU environment, such as common treatments provided, needed decision making, and difficulties related to accessing physicians and nurses. The hypothetical scenario was shown to the participants as follows:

Imagine that your family member is admitted to an ICU. Currently, he/she is unconscious. You are staying in a waiting room next to the ICU and allowed to meet the family member for only 30 minutes twice a day, as per the hospital policy. Now, you are confused about the patient's condition and anxious about what will happen to the patient. You are also not sure if all the life‐sustaining treatments are meaningful to the patient and are worried about the patient's suffering. You are not aware what you can do for the patient.

In the ICU, there is a certified critical care advanced practice nurse. To be certified, a nurse should have a master's degree at the specialty of critical care and at least three years of clinical experience in critical care settings. This critical care advanced practice nurse provides education and counselling service to help you with an update about patient's condition, explain each treatment given to the patients and how the patient is responding and gives information about what the family could do for patient's comfort. Moreover, the nurse provides information about advance care planning and supports yours and your family's decision making. Would you be willing to pay for this service?

The next section of the questionnaire asked about respondents' preferences in paying for the service. The elicitation method of this study was a double‐bounded dichotomous choice. Therefore, on the basis of the given scenario, the participants were asked to decide whether they were willing to pay at the bid price proposed. The initial bid price was 20 000 KRW (16.51 USD), 30 000 KRW (24.77 USD), 40 000 KRW (33.02 USD), or 50 000 KRW (41.28 USD) (Table 1). These initial bid prices were established on the basis of a pretest with 40 respondents that included the following open‐ended question: “How much will you pay for this service?” The first bid prices were based on the prices at the 20%, 40%, 60%, and 80% levels of the cumulative distribution from the pretest (Kim, Kim, & Kang, 2010; Kwon & Ryu, 2013). One of these four initial bid prices was randomly assigned to each participant.

**Table 1:** Design of first bid price

Cumulative Distribution	Bid Price
20%	16.51 USD (20 000 KRW)
40%	24.77 USD (30 000 KRW)
60%	33.02 USD (40 000 KRW)
80%	41.28 USD (50 000 KRW)

*Note*. 1 USD = 1211.12 KRW, the average exchange rate in 2016.

A double‐bounded dichotomous choice format was used as it has been reported that the double‐bounded method is asymptotically more efficient than the single‐bounded method (Hanemann, Loomis, & Kanninen, 1991). The double‐bounded method asks a second dichotomous choice question depending on the answer to the first dichotomous choice question, while the single‐bounded method asks just one dichotomous choice question. In this study, if a participant answered “yes” to the initial bid, the following question asked whether he or she was willing to pay double the first bid. On the other hand, if a participant answered “no” to the initial bid, the following question asked whether he or she was willing to pay half the first bid.

The last part of the questionnaire dealt with socio‐economic information. Questions about gender, age, education, and household income were asked. These characteristics have been reported as influencing WTP Hanemann, 1984; Kwon & Ryu, 2013).

### 2.5 Ethical consideration

This study was conducted according to the Declaration of Helsinki in 1995. The study participants were members who enrolled in the online panel of the company. A dataset without personal identifiers provided by the company was utilized for the analysis. The researchers and the survey company entered into a contract. Informed consent was obtained online from the participants, and individuals who consented to participate in the study completed the questionnaires for the survey.

### 2.6 Statistical analyses

To distinguish protest zero bids from true zero bids, the participants were asked about their reason for refusing any payment via a multiple‐choice question. A zero bid is considered a protest zero bid when a respondent does not want to pay for reasons associated with the process of contingent valuation itself, while a bid is considered a true zero bid when a respondent does not want to pay because he or she would not obtain any benefit from the nonmarket resource. Protest zero bids occur, for example, when respondents perceive that paying extra for nonmarket resources is unfair. Thus, these protest zero bids were removed from the analysis (Jorgensen, Syme, Bishop, & Nancarrow, 1999). In this study, protest zero bids were identified by a follow‐up question that asked the respondents why they did not want to pay: “Please specify the reason(s) for your unwillingness to pay for this education program.” Respondents who chose the response “this service should be covered by the National Health Insurance” or the response “this service should be included in the ICU admission fee that is currently covered by the National Health Insurance” were considered to have made protest zero bids (Lo & Jim, 2015; Shafie, Lim, Chua, Hassali, & A., 2014). After excluding protest zero bids, data from 496 respondents were utilized for the probit model analysis. The protest zero bid rate was 19.3%, and protest zero bid rates in previous CVM research ranged from 4% to 56% (Meyerhoff et al., 2014).

A medium WTP value was calculated on the basis of Hanemann's logit model using the maximum likelihood method (Hanemann, 1984). Hanemann (1984) stated that the median is relatively robust, while the mean is extremely sensitive to changes in the shape of the distribution. This study utilized the median value of WTP. The following equation was utilized:

$$\delta_{0}+\delta_{1}\ln E^{*}=0,\text{or}\;E^{*}=e^{-\delta_{0}/\delta_{1}},$$

where *E* ^*^ is the median of an individual's maximum WTP (Hanemann, 1984, p. 339).

For the logistic regression, dummy codes were created for some covariates, including gender (male: 0; female: 1), education level (lower than college: 0; college or higher: 2), and household income (lower than 5 million KRW/month: 0; 5 million KRW/month or higher: 1).

## 3 RESULTS

### 3.1 General characteristics of the participants

The mean age of the participants was 32.5 years (SD = 8.053), and there were 258 (52.1%) males and 237 (47.9%) females. The participants' socio‐economic characteristics are summarized in Table 2.

**Table 2:** General characteristics of the study participants (N = 495)

Variables		N (%)	Mean (SD)
Gender	Male	258 (52.1)	
	Female	237 (47.9)	
Age			32.5 (8.053)
Education level	Lower than college level	128 (25.9)	
	College level or higher	367 (74.1)	
Household income per month	Lower than 5 million KRW (4128.41 USD)	337 (68.1)	
	5 million KRW (4128.41 USD) or higher	158 (31.9)	

### 3.2 WTP for critical care APN services

#### 3.2.1 Factors associated with the public's WTP

Table 3 shows the results of the logistic regression model. The model was significant (*χ* ^2^ = 210.874, *df* = 1, *P* < .001). The first bid had the greatest influence on WTP. Age and income were also significantly associated with WTP. Younger participants were more likely to be willing to pay. Moreover, participants who had a household income of at least 5 million KRW (4128.41 USD) per month were more likely to be willing to pay than those who had a household income of less than 5 million KRW (4128.41 USD) per month.

**Table 3:** Results of the logistic regression model

	*B*	S.E.	Wald	*df*	Sig.	Exp(*B*)
lnbid	−1.264	0.094	181.759	1	<.001	0.282
Gender	−0.039	0.114	0.115	1	.735	0.962
Age	−0.018	0.008	5.321	1	.021	0.982
Education level	0.111	0.142	0.606	1	.436	1.117
Income	0.309	0.123	6.337	1	.012	1.363
Constant	13.914	1.019	186.451		<.001	1 103 532.368
−2 Log likelihood	1818.824					
Cox and Snell *R* ^2^	0.132					
Nagelkerke *R* ^2^	0.178					

#### 3.2.2 Estimation of median WTP

Table 4 shows the results for the estimation of median WTP, and the equation used for the WTP calculation is shown below. The median WTP was 43 111.74 KRW (35.60 USD).

**Table 4:** Prediction of the probability of willingness to pay

		*B*	Mean	*B* × Mean
β	lnbid	−1.264		
α	Gender	−0.039	0.52	−0.02028
	Age	−0.018	32.55	−0.5859
	Education level	0.111	0.74	0.08214
	Income	0.309	0.32	0.09888
	Constant	13.914		13.914
Total				13.48884
WTP (median) = $e^{-\frac{\delta 0}{\delta 1}}=e^{-\frac{13.48884}{-1.264}}=43111.74\;\mathrm{KRW}=35.60\;\mathrm{USD}$				

Abbreviation: WTP, willingness to pay.

## 4 DISCUSSION

### 4.1 WTP for critical care APNs' education services

In this study, the median WTP was 43 112 KRW (35.60 USD). It is difficult to compare the medium WTP value in this study with WTP values reported in previous studies because of the different characteristics of the services examined in the various studies. Most previous studies utilizing CVM for health care services focused on medical tests or treatments such as diagnostic tests, ovulation induction treatment, implantable cardioverter‐defibrillator therapy, hypertension, myocardial infarction, and implants (Garni et al., 2012; Lin et al., 2013; Nowakowska et al., 2011; Poder, He, Simard, & Pasquier, 2014; Yasunaga et al., 2006).

In a similar study, Martín‐Fernández and his colleagues utilized CVM to examine the monetary value of a service provided by nurses in Spain. In their study, the median WTP for a nursing consultation during primary care was 10 EUR (9.23 USD). This value seems to be much lower than the value of 43 112 KRW (35.60 USD) observed in the current study. However, each participant in the study by Martín‐Fernández et al. (2013) was a patient who visited one of 23 health centres with a health problem and responded on the basis of his or her real experience with a nurse. Therefore, the participants' perceptions of the consultation duration varied, ranging from less than 1 minute to more than 30 minutes. Approximately 60% of the participants in that study perceived the consultation duration to be 15 minutes or less.

On the other hand, the present study revealed that higher income and younger age were associated with higher WTP. Previous studies have consistently reported that higher income is associated with higher WTP in health care services (Lin et al., 2013; Nowakowska et al., 2011; Shafie, Hassali, & A., 2013). Age was negatively associated with WTP in this study, which is consistent with the results of other WTP studies (Hou et al., 2014; Niringiye & Omortor, 2010). These results might reflect the fact that higher income and younger age are factors related to paying premiums for better quality health care services than those provided by the National Health Insurance in South Korea (Baek, Park, & Byun, 2012).

### 4.2 Education services in the National Health Care System in South Korea

Currently, 11 types of education services are included in the list of benefits according to the National Health Insurance Act: education for cancer management, heart disease management, stoma care, chronic renal failure management, diabetes management, hypertension management, hyperlipidaemia management, aplastic anaemia management, caries prevention, inborn errors of metabolism management, and epilepsy management. In addition, the government has undertaken a pilot programme to include preoperative and postoperative patient education in the benefit system (Ministry of Health and Welfare, 2018).

According to the National Health Insurance Act, the government publicizes the list of health care benefits, which implies legally approved medical practices. The list of health care benefits has two parts: insured benefits and uninsured benefits. A service that is not included under either the insured or uninsured benefits should not be charged to the National Health Insurance or to the patient's uninsured benefits because the service is considered unauthorized medical care (Kim et al., 2011). When the treatment is on the list of insured benefits, the National Health Insurance pays for most of the treatment, and the patient pays for a small portion through a copayment. The price of each insured benefit is fixed nationally. When the treatment is on the list of uninsured benefits, the patient is responsible for paying for all of the treatment. The prices of uninsured treatments are determined by the hospital; thus, prices vary by hospital. However, the prices of uninsured benefits should be reported to the government and are posted on the governmental website and accessible to the public (Lee, 2013).

Of the 11 education services included in the list of benefits, only four are insured services: education for cancer management, heart disease management, stoma care, and chronic renal failure management. The prices of these services ranged from 20 660 KRW (17.05 USD) to 94 470 KRW (78.00 USD) as of 1 January 2018, depending on various factors such as type of disease or health problem, specific content of the education service, and location where the education service is provided (clinic, hospital, or tertiary hospital) (Health Insurance Review and Assessment Service, 2018).

Given the prices of insured education services under the National Health Insurance System, the median WTP observed in this study, 43 112 KRW (35.60 USD), seems quite realistic. For example, one type of education offered for cancer management involves a 30‐minute education and counselling service about chemotherapy. The rate for this service in a tertiary hospital is 42 360 KRW (34.97 USD) (Health Insurance Review and Assessment Service, 2018), which is similar to the median WTP observed in this study. This cancer education service is similar to the hypothetical critical care APN education service examined in the present study in terms of the duration of the education service and the provider. According to the National Health Insurance Act, an oncology APN or physician could provide this type of cancer education. However, we are limited in our ability to directly compare the rates of the two education services because the content of the services and the targeted learners are very different.

### 4.3 The education role of critical care APNs

Education and counselling is one of the advanced practice nursing competencies designated by the Korean Accreditation Board of Nursing Education (Korean Accreditation Board of Nursing Education, 2004). In ICUs, nurses and physicians regard education and counselling as a major role of critical care APNs (Sung, Yi, Kwon, & Cho, 2006). However, hospitals seem to consider the education and counselling services provided by critical care APNs nonproductive services in terms of financial reimbursement (Kim et al., 2011) because hospitals cannot be reimbursed for these specific education and counselling services under the fee‐for‐service model of the National Health Insurance System. If the education and counselling services provided APNs were included in the list of benefits under the National Health Insurance Act, then hospitals might support providing time and resources for APNs to engage in these education and counselling services.

In this study, Korean people recognized the economic value of an education service provided by critical care APNs. Nurses should make an effort to influence health policy to include education and counselling services provided by APNs in the health care fee‐for‐service system. Notably, a pilot programme was recently undertaken regarding the inclusion of preoperative and postoperative patient education and counselling in the benefit system (Ministry of Health and Welfare, 2018) because of the consistent efforts of surgery societies (Yang, 2018). Thus, professional nursing groups need to use their voice to influence policy. In particular, the Korean Nurses Association is expected to show leadership to advocate for including APN services in the benefit system. A member of the Korean Nurses Association services serves as a committee member on the Health Insurance Policy Deliberation Committee, which makes major decisions about health insurance benefits and the pricing for each service.

### 4.4 Limitations

First, this study used an Internet‐enabled panel, which may have introduced issues related to access to populations (Wright, 2005). Although 98.8% of households in South Korea have access to the Internet (OECD, 2018), placing South Korea among the top OECD countries with regard to Internet access, there are some disparities in Internet usage rates depending on age and region. The average rate of Internet usage for all ages is 83%, while it is only 32% for individuals aged 60 years or older (Statistics Korea, 2015). This study included young and middle‐aged adults who had Internet access and were capable of filling out a web‐based survey. Therefore, this study might have limited generalizability.

Second, the data in this study were collected from a pre‐recruited panel, which could have introduced sampling bias (Sueki, 2016). Moreover, the study participants were members of the general public who resided in the community; therefore, the majority may not have had any experience with the environment of an ICU.

As with other CVM studies, this study utilized a specific hypothetical scenario that described the environment of ICUs. When interpreting the results of this study, the fact that WTP was estimated under this condition should be considered. Further CVM studies could be conducted with family members caring for a patient in the ICU to examine their real experiences with the care provided in ICUs.

Finally, a scenario about an education and counselling service programme was given to the survey respondents. Because there is no standardized form for such a programme in terms of duration and content in the real health care system, a hypothetical programme was created for this study, and WTP was based on that hypothetical scenario. The respondents reported their WTP considering elements of the scenario such as duration and content of the programme. Therefore, this monetary value should be considered in the context of such a scenario.

## 5 CONCLUSION

This study captured the economic value of a critical care APN education and counselling service that is not offered under the current health care benefit system. The WTP value observed in this study seems to be realistic compared with the prices for other education and counselling services currently covered by the Korean National Health Insurance. Nursing societies and nurses' associations should work together to expand the role of APNs by including their services in the list of benefits covered by the fee‐for‐service system of the Korean National Health System.

## CONFLICT OF INTEREST

No conflict of interest exists.

## AUTHORSHIP STATEMENT

All authors (Dr. Ko, Dr. Koh, and Dr. Kwon) participated in each of the following steps together: designing the study, collecting the data, analysing the data, preparing the manuscript, and approving the final version for submission.
